# Dataset of road surface images with seasons for machine learning applications

**DOI:** 10.1016/j.dib.2022.108023

**Published:** 2022-03-08

**Authors:** Sonali Bhutad, Kailas Patil

**Affiliations:** Vishwakarma University, Pune, India

**Keywords:** Road surface monitoring, Sustainable transportation, Pothole detection, Object detection, Computer vision

## Abstract

Road surface monitoring plays a vital role in ensuring safety and comfort for the various road users, from pedestrians to drivers. Furthermore, this information is useful for the maintenance of the roads. The road condition deteriorates due to volatile weather. Thus the main objective of the proposed paper is to create an image dataset of the road surface for two seasons, i.e. summer and rainy. Accordingly, we created road surface images for different roads such as paved and unpaved roads. These folders consist of two subfolders for Rainy and Summer potholes. The dataset consists of 8484 images and 10 videos. This dataset is highly useful for machine learning experts working in the field of automatic vehicle controlling and road surface monitoring.

## Specifications Table

**Table:** 

Subject	Computer Vision and Pattern Recognition
Specific subject area	Road Surface Detection
Type of data	Image, Video
How datapoints were acquired	Road surface images in different forms such as damaged road surface, speed breaker and road surface with water and without water were considered for the dataset. Images were captured using Samsung Galaxy A22 Quad camera with the specifications as below, 48 MP, f/1.8, (wide), 1/2.0″, 0.8 µm, PDAF, OIS 8 MP,f/2.2,123˚(ultrawide),1/4.0″,1.12 µm 2 MP,f/2.4,(macro) 2 MP, f/2.4, (depth)
Data format	Raw
Parameters for data collection	The dataset is composed of 8484 RGB images (512 × 512) pixels, horizontal 96 dpi, vertical 96 dpi) in .jpg format.
Description of data collection	The collection of the image dataset was done in-field, at day-light during varying sunlight. Images represent the top view and side view of the road surface.
Data source location	City/Town/Region: Nashik and Mumbai Country: India Latitude and longitude samples/data: 19.9975° N, 73.7898° E, 19.0760° N, 72.8777° E
Data accessibility	Repository name: Dataset of Unpaved and Paved Road Surface with Seasons Data identification number: doi: 10.17632/tj2m7zz4rg.2 Direct URL to data: https://data.mendeley.com/datasets/tj2m7zz4rg/2

## Value of the Data

• This dataset is essential as it contributes to the future applications of the Sustainable Development Goal-11 of the United Nations, i.e., ``sustainable-transport'' (UN SDG -11) [3]. We concentrated on the forms of road surface available during different seasons to achieve accuracy in road surface detection, which makes this dataset unique.
• These datapoints are available in the public repository for all the Research Institutes, Scientific communities and Policymakers.
• This is the first dataset that provides road surface images according to seasons that could be used in a wide variety of future research studies related to road accident prevention and surface inspection [4].
• The data may be reused for conducting experiments related to road accident prevention, Pothole detection, road surface water detection, automatic vehicle controlling mechanism and path navigation mechanism. Moreover, the researchers involved in earth surface inspection may benefit indirectly [2].

## 1 Data Description

In transportation infrastructure, one of the main concerns is potholes in roads [1]. Most machine learning techniques for autonomous driving are trained on data collected in certain environments and are not reliable in cross weather conditions [2,6]. Hence we created the road surface dataset with seasons. The dataset folder comprises separate folders for paved and unpaved roads. Further, they are divided into subfolders. The images in each subfolder have been categorized into Final, Raw and Rotated folders. The Raw data folder images are unprocessed; hence resolution range is given for them (Table 2, Point.6). The images in the other folders were resized and further rotated by 90°. The image count is provided according to the seasons, type of road and class of the dataset images (Table 4).

The dataset contains 8484 images and 10 videos. Every image and video were stored in .jpg format and MP4 format, respectively. In the road surface dataset, different structures were considered, such as (a) Pothole in rains on the paved and unpaved road, (b) Pothole in summer on the paved and unpaved road (c) Speed breaker and (d) Roadside Barriers. Cement concrete and tar roads were considered for the paved road condition, while roads made of only soil and stones were unpaved. All the images were taken from the top view and the side view. These images were captured during 2020 and 2021 from two cities, namely Nashik and Mumbai, of Maharashtra, India.

Based on the road structure, the directory structure is divided into three main folders: (1) Paved Road, (2) Unpaved and (3) Video. The first two folders are further divided into subcategories according to the status of the pothole during summer and rains. Along with potholes, we also added speed breaker and roadside barrier images. The video folder consists of videos according to seasons for various road surface types (Fig. 2).

## 2 Experimental Design, Materials and Methods

### 2.1 Experimental design

The image data acquisition process is shown in Fig. 3. The Road surface images were acquired using the Samsung Galaxy A22 mobile's high-resolution quad rear camera. In all, 8484 images were captured using a camera and then were segregated and saved in respective folders as per the Road type and class of the image (Fig. 2). The 10 videos were captured for various road surfaces and stored in the Video folder.

The dataset is collected for the images available in two seasons, i.e. Rainy and Summer. Therefore, the duration from April to December is chosen for capturing images (Table 1).

**Table 1:** Data acquisition requirement.

SI. No.	Year	Month	Season	Frequency	Activity
1	2021	April-December	Rainy and Summer	Daily	Captured Images in the morning, afternoon, evening, and late evening
2	2020	May - November	Rainy and Summer	Daily	Captured Images in the morning and afternoon

**Fig. 1:**
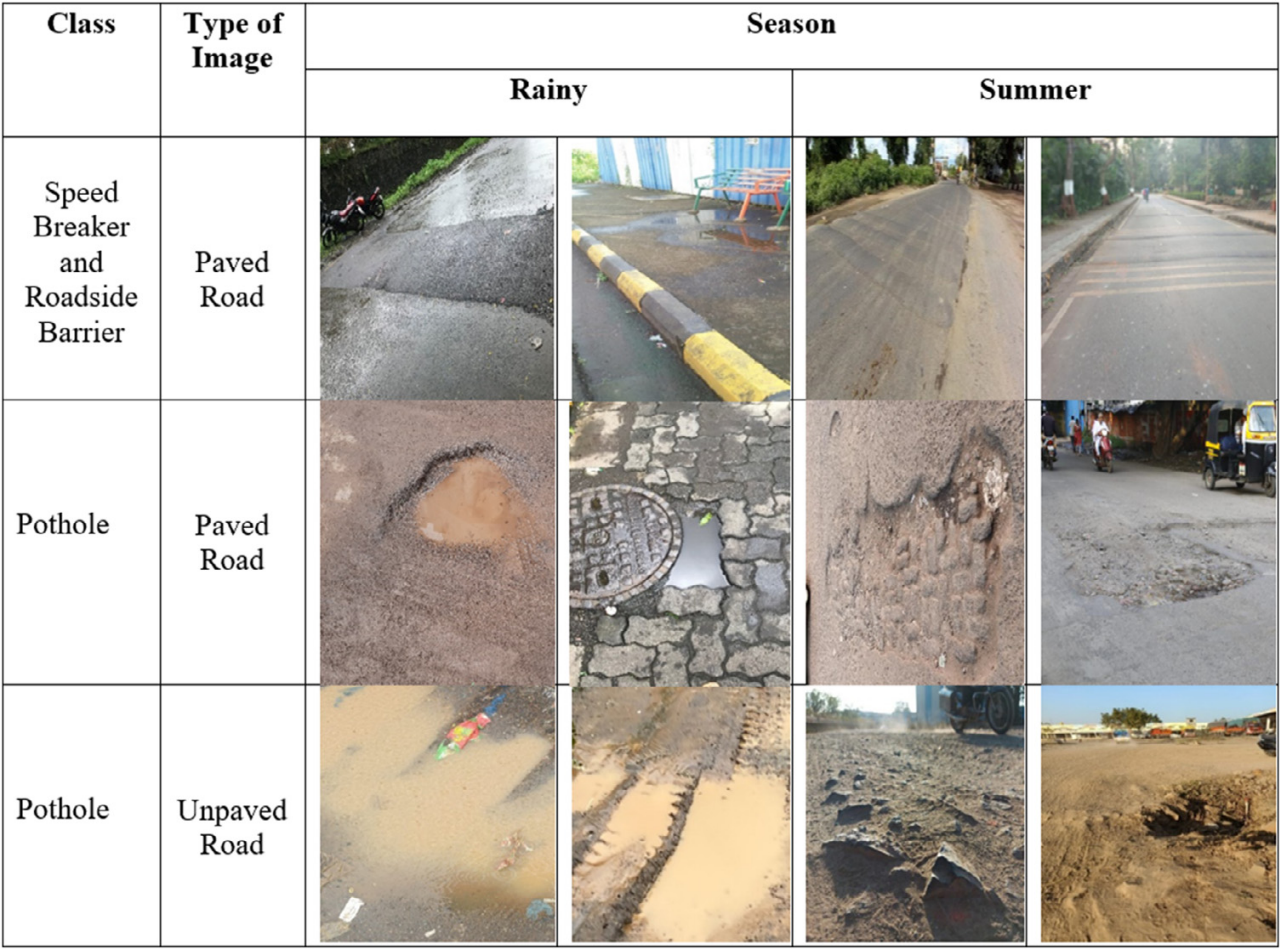
Partial images of the dataset.

The road surface was available in different forms such as speed breakers, potholes and roadside barriers (Fig. 1). Thus, the dataset was prepared by considering all adverse conditions required for Road surface detection according to season. To cope with adverse conditions, it is necessary to collect images in different illuminations. Hence the images were captured in the morning, afternoon, evening, and late evening from the top and the side view (Table 4).

**Fig. 2:**
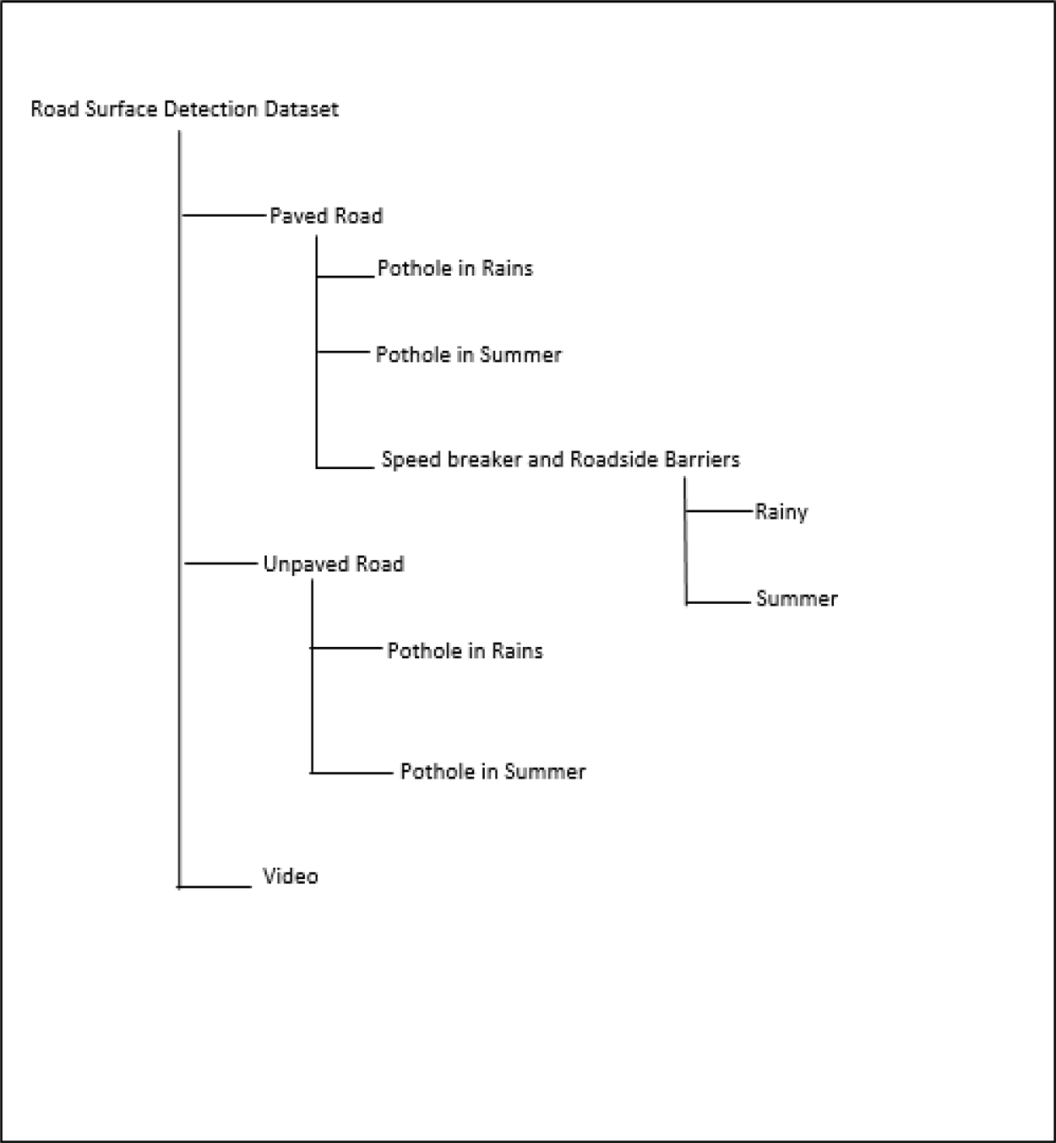
Dataset directory structure.

**Fig. 3:**
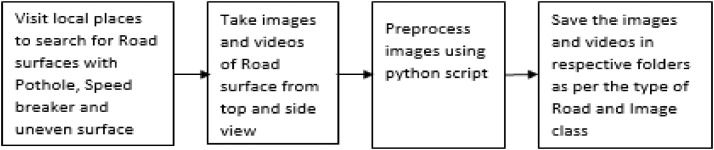
Road surface data acquisition process.

### 2.2 Materials or specification of image acquisition system

The road images were captured using the Samsung Galaxy A22 RGB Quad camera (Table 2) [5]. A 15 W battery was used to power all the components of the imaging system. All dataset images were resized to 512 × 512 dimensions using python script (Table 3). The images are in .jpg format, and the videos are in mp4 format. Due to the complexities of earth surface and remote sensing data, it is necessary to identify road surfaces in various conditions [1]. Thus to overcome the time-dependent and weather variations of illumination in an outdoor environment, the dataset consists of road surface images in the form of speed breaker, uneven road surface, potholes in rains and potholes in summer.

**Table 2:** Specification of image acquisition system.

Sr. No.	Camera Particulars	Details
1	Camera makers	Samsung
2	Camera model	Samsung Galaxy A22
3	F-stop	f/1.8, f/2.2, f/2.4,f/2.4 aperture
4	Exposure time	1/33 s
5	Flash mode	No flash mode
6	Image resolution	Min-300 × 204 Max-4128 × 2322

**Table 3:** Specification of images.

		Details as per Road Classes	
Sr. no	Particulars	Paved Road	Unpaved Road
1	Dimension	512 × 512	512 × 512
2	Width	512 pixel	512 pixel
3	Height	512 pixel	512 pixel
4	Horizontal Resolution	96 dpi	96 dpi
5	Vertical Resolution	96 dpi	96 dpi
6	Bit Depth	24	24

### 2.3 Method

Table 4 describes the classes, number of images taken and the environments in which images are taken. A handheld mobile camera was used to capture images from the top view and side view. The images were captured at a man's height by bending down. The images of speed breakers, roadside barriers and potholes during different seasons for paved and unpaved roads were included.

**Table 4:** Road surface dataset details.

Class	Season	Direction of Image coverage	Type	Time of Image Coverage	Count
Uneven Road	Summer	Top view Side view	Paved Road-553	Morning, Afternoon, Evening, and Late Evening	553
Speed Breaker	Summer	Top view Side view	Paved Road-440	Morning, Afternoon, Evening, and Late Evening	440
Pothole with water	Rainy, Summer	Top view Side view	Paved Road-1564 Unpaved Road-760	Morning, Afternoon, Evening, and Late Evening	2324
Pothole without water	Rainy, Summer	Top view Side view	Unpaved Road-118	Morning, Afternoon	118
Original Images					4242
Rotated Images					4242
Total Images					8484

## Ethics Statement

This data is available in the public domain, and no funding is received for the present effort. There is no conflict of interest.

## CRediT authorship contribution statement

**Sonali Bhutad:** Methodology, Data curation, Formal analysis, Writing – original draft. **Kailas Patil:** Conceptualization, Writing – review & editing, Supervision, Project administration.

## Declaration of Competing Interest

The authors declare that they have no known competing financial interests or personal relationships which have or could be perceived to have influenced the work reported in this article.
